# Early-Onset Leukonychia as a Diagnostic Clue in Familial Hailey-Hailey Disease

**DOI:** 10.7759/cureus.96310

**Published:** 2025-11-07

**Authors:** Assia El Bouhmadi, Hanane Rachadi, Bouchra Baghad, Fatima Zahra El Fatoiki, Soumiya Chiheb

**Affiliations:** 1 Dermatology, Ibn Rochd University Hospital Center, Faculty of Medicine and Pharmacy of Casablanca, Hassan II University, Casablanca, MAR

**Keywords:** genodermatosis, hailey-hailey, hereditary dermatological disorders, leukonychia, nail disorders

## Abstract

Hailey-Hailey disease (HHD) is a rare autosomal dominant genodermatosis characterized by recurrent intraepidermal blistering and erosions, most often affecting flexural and intertriginous areas. Although its clinical presentation is distinctive, the disease exhibits considerable variability and is often misdiagnosed, particularly in early or atypical forms. Nail involvement, historically considered absent, has recently been recognized as an underappreciated feature that may provide valuable diagnostic clues.

We report a familial case of HHD spanning three generations, in which a single longitudinal leukonychia in the proband preceded the onset of typical skin lesions. The initial nail change, unassociated with trauma or systemic disease, prompted a detailed family history revealing recurrent erosive lesions in relatives. Months later, the proband developed painful, fissured, erythematous plaques in flexural regions, confirmed histologically as HHD. Treatment with topical calcineurin inhibitors, oral doxycycline, and ultraviolet B (UVB) phototherapy led to marked clinical improvement and reduced flare frequency, although the leukonychia remained unchanged.

This case highlights the diagnostic importance of subtle nail findings in HHD. Early-onset or isolated leukonychia, often asymptomatic and easily overlooked, may precede cutaneous involvement and thus represent a valuable marker for early detection in at-risk individuals. Recognition of such signs can facilitate timely diagnosis, genetic counseling, and preventive management within affected families. Nail alterations in HHD appear long-lasting and independent of disease activity, underscoring their potential role as a stable phenotypic manifestation of this disorder.

Early identification of these subtle nail changes can therefore improve patient outcomes by guiding early therapeutic intervention and reducing the delay in diagnosis often associated with this underrecognized condition.

## Introduction

Hailey-Hailey disease (HHD), also known as chronic familial benign pemphigus, is a rare autosomal dominant genodermatosis caused by mutations in the ATP2C1 gene. The resulting defect in keratinocyte adhesion leads to recurrent intraepidermal blistering and erosions, classically affecting flexural and intertriginous areas [1].

Although clinically distinctive, HHD shows wide phenotypic variability, even within the same family. The disease is chronic, relapsing, and significantly impacts patients’ quality of life.

Historically, nail involvement was thought to be absent in HHD, unlike in Darier’s disease. However, Burge and later Kirtschig et al. emphasized that nail changes - most commonly white longitudinal bands - can occur in up to 70% of patients but are often overlooked unless specifically examined [2,3]. These subtle findings may serve as valuable diagnostic clues, particularly in early or atypical presentations.

We report a familial case of HHD across three generations, in which a single longitudinal leukonychia in the proband preceded the onset of typical cutaneous lesions, leading to the diagnosis and recognition of the disorder within the family.

## Case presentation

**Figure 1:**
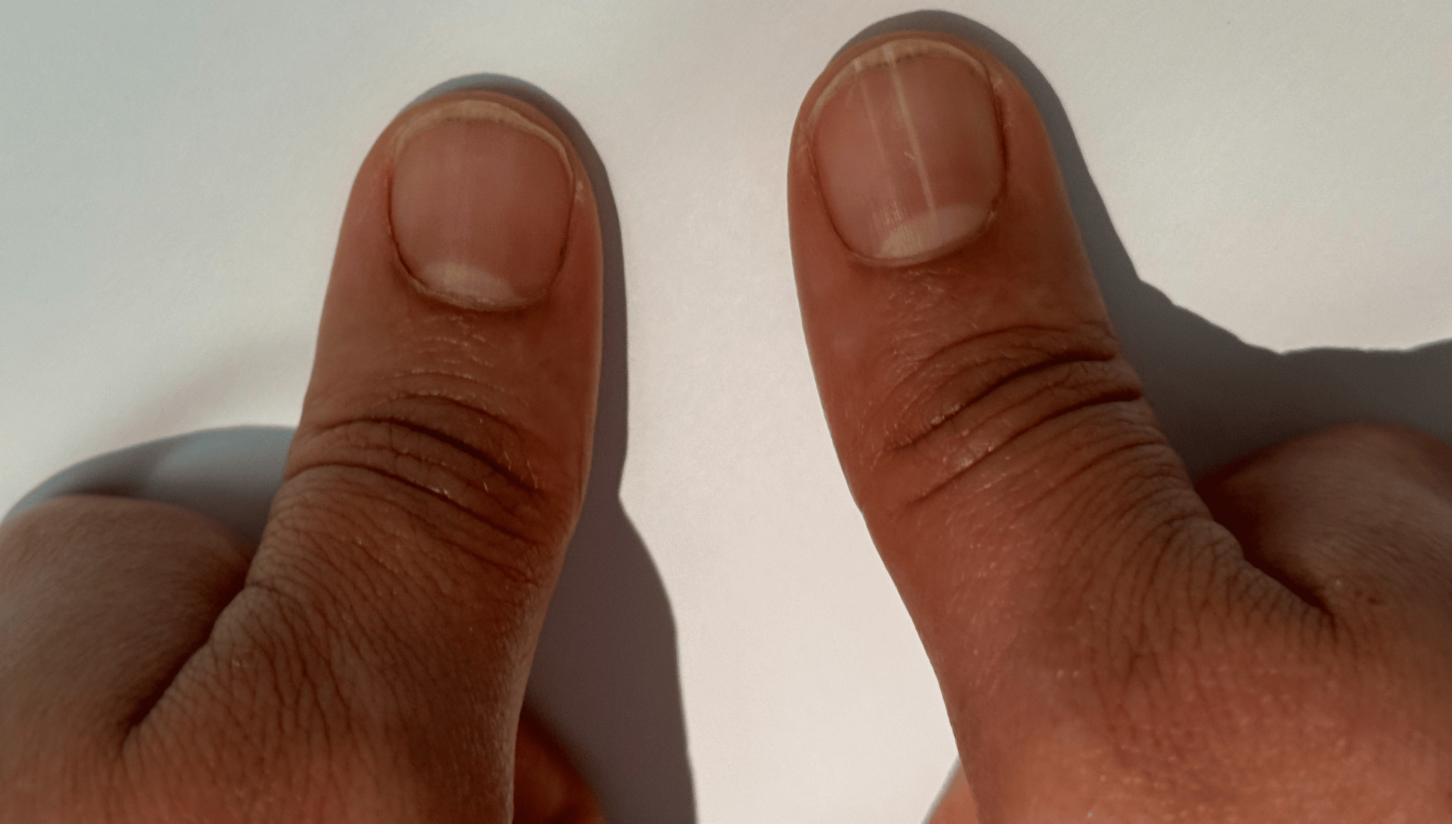
Longitudinal leukonychia on the right thumbnail

A 35-year-old woman consulted for a single longitudinal white band on her thumbnail (Figure 1), present for several months, without any skin lesions at the time. No trauma or systemic disease could explain the nail change. Given its unusual presentation, a detailed family history was obtained. Her mother, grandmother, and younger sister had a history of recurrent erosive lesions in flexural areas, though none had nail abnormalities.

**Figure 2:**
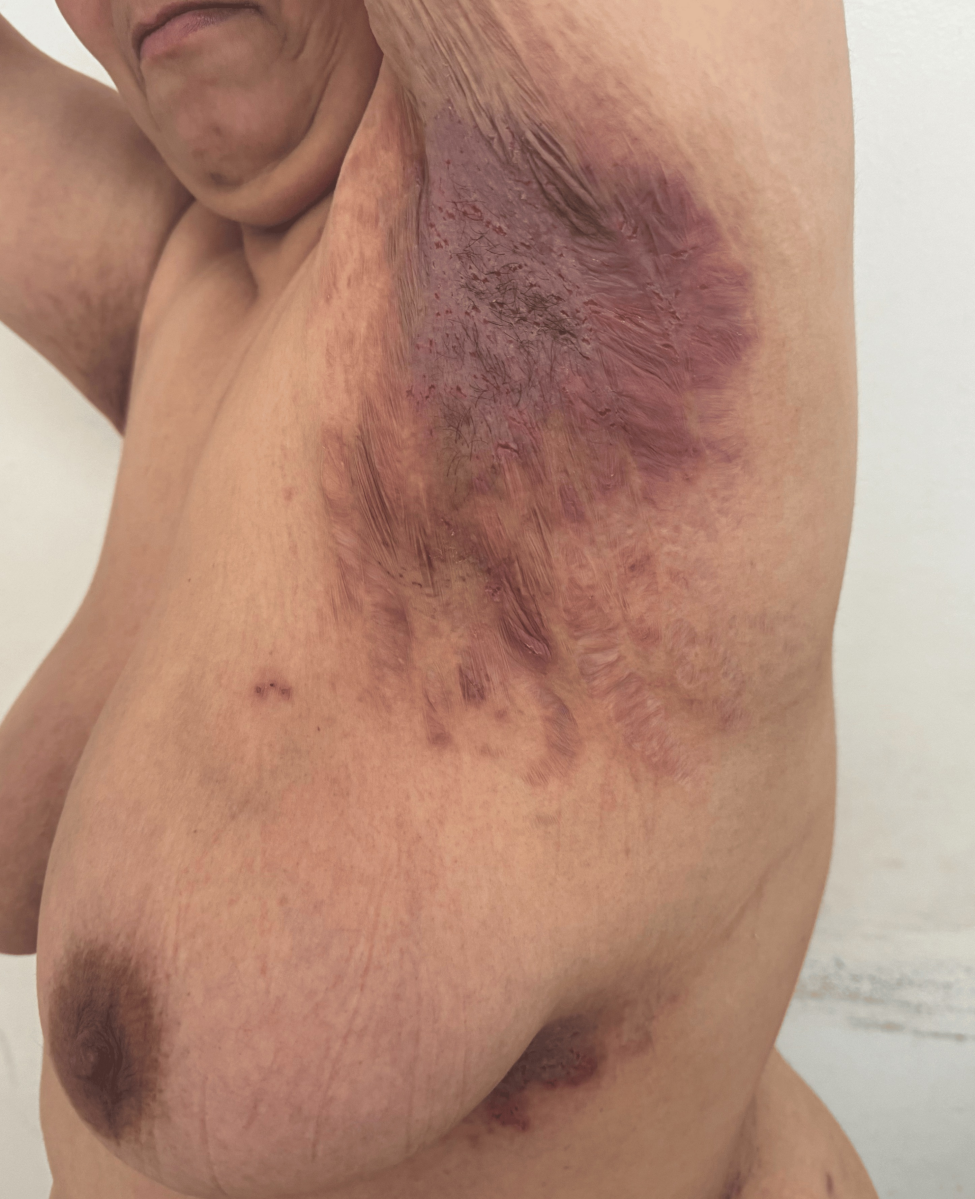
Symmetrical fissured plaques involving the axillary folds

**Figure 3:**
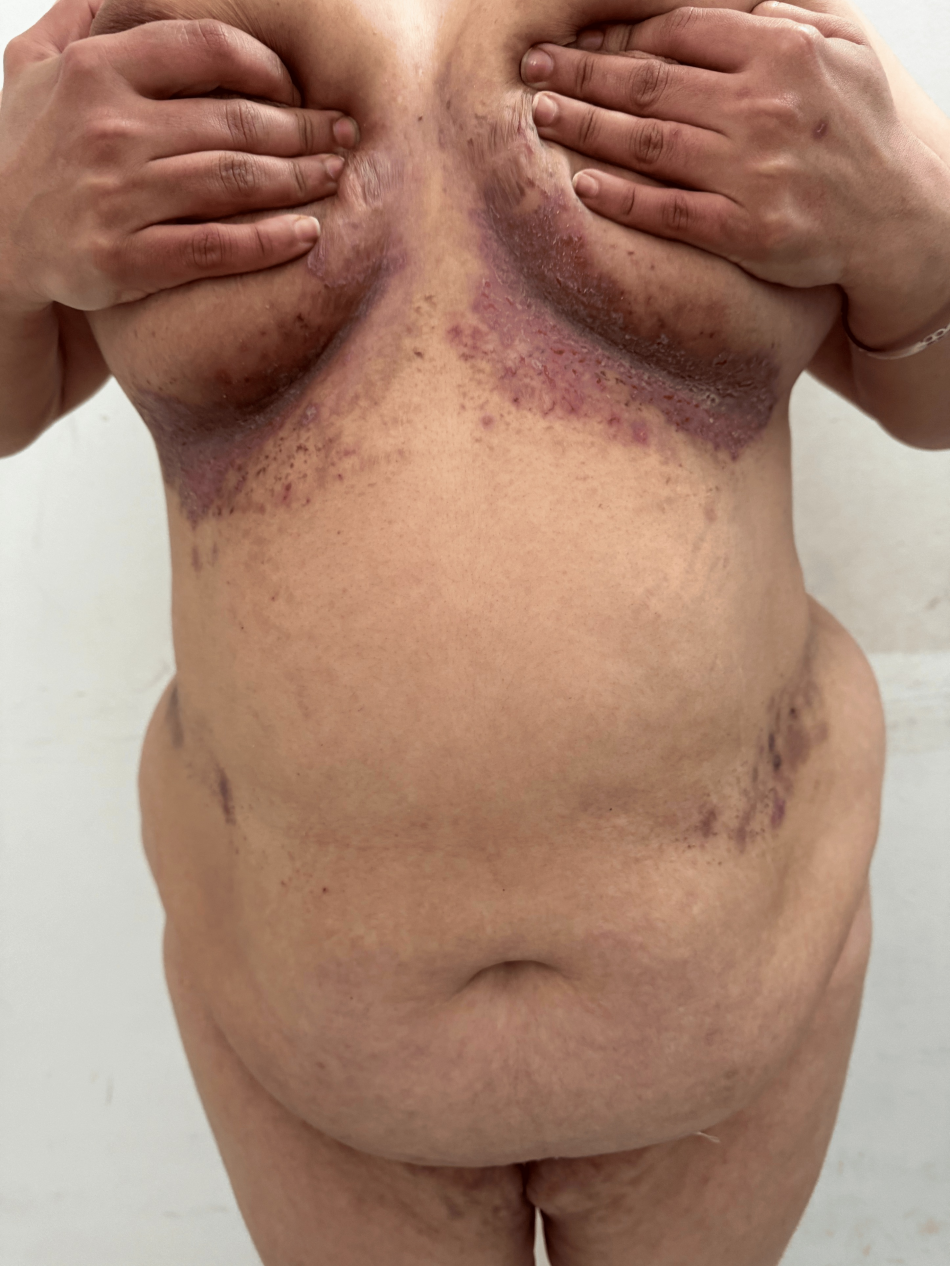
Macerated erythematous plaques with linear erosions in the inframammary region and in the abdominal folds

Several months after the initial consultation, the proband developed painful erosive plaques in the axillary, inguinal, and inframammary folds, consistent with HHD, which she initially treated with over-the-counter topical corticosteroids. Examination revealed symmetrical fissured plaques involving the axillary and inguinal folds (Figure 2), as well as macerated erythematous plaques with linear erosions in the inframammary region and in the abdominal folds (Figure 3). The patient reported intense pruritus and burning sensations, significantly impairing her quality of life. The mucous membranes were unaffected. A persistent longitudinal leukonychia was noted on a single fingernail, without additional nail abnormalities. Histopathology confirmed suprabasal acantholysis with a dilapidated brick wall appearance, consistent with HHD. Direct immunofluorescence was negative, ruling out autoimmune bullous disease.

**Figure 4:**
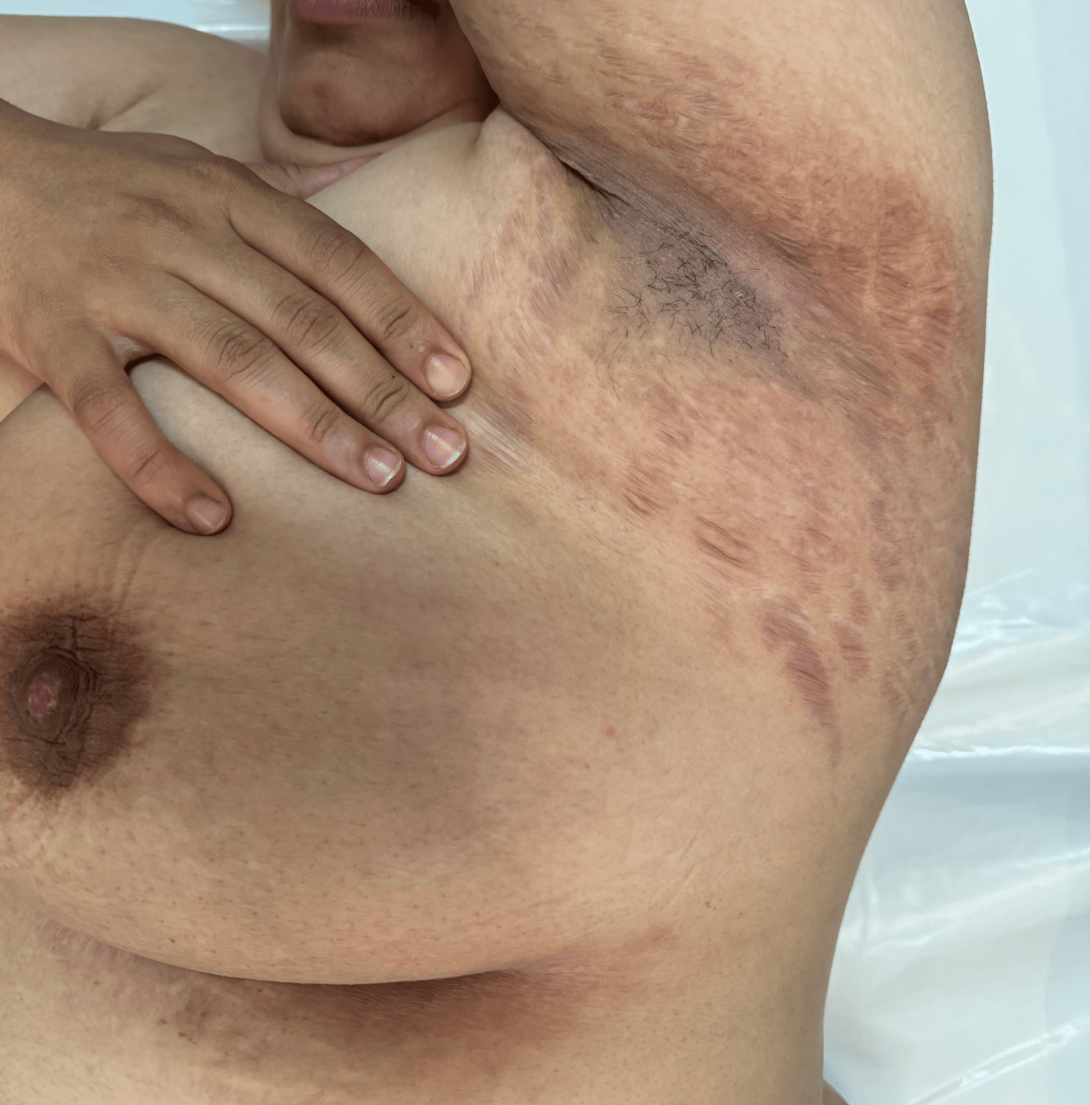
Clinical aspect of the axillary fold after three months of treatment

**Figure 5:**
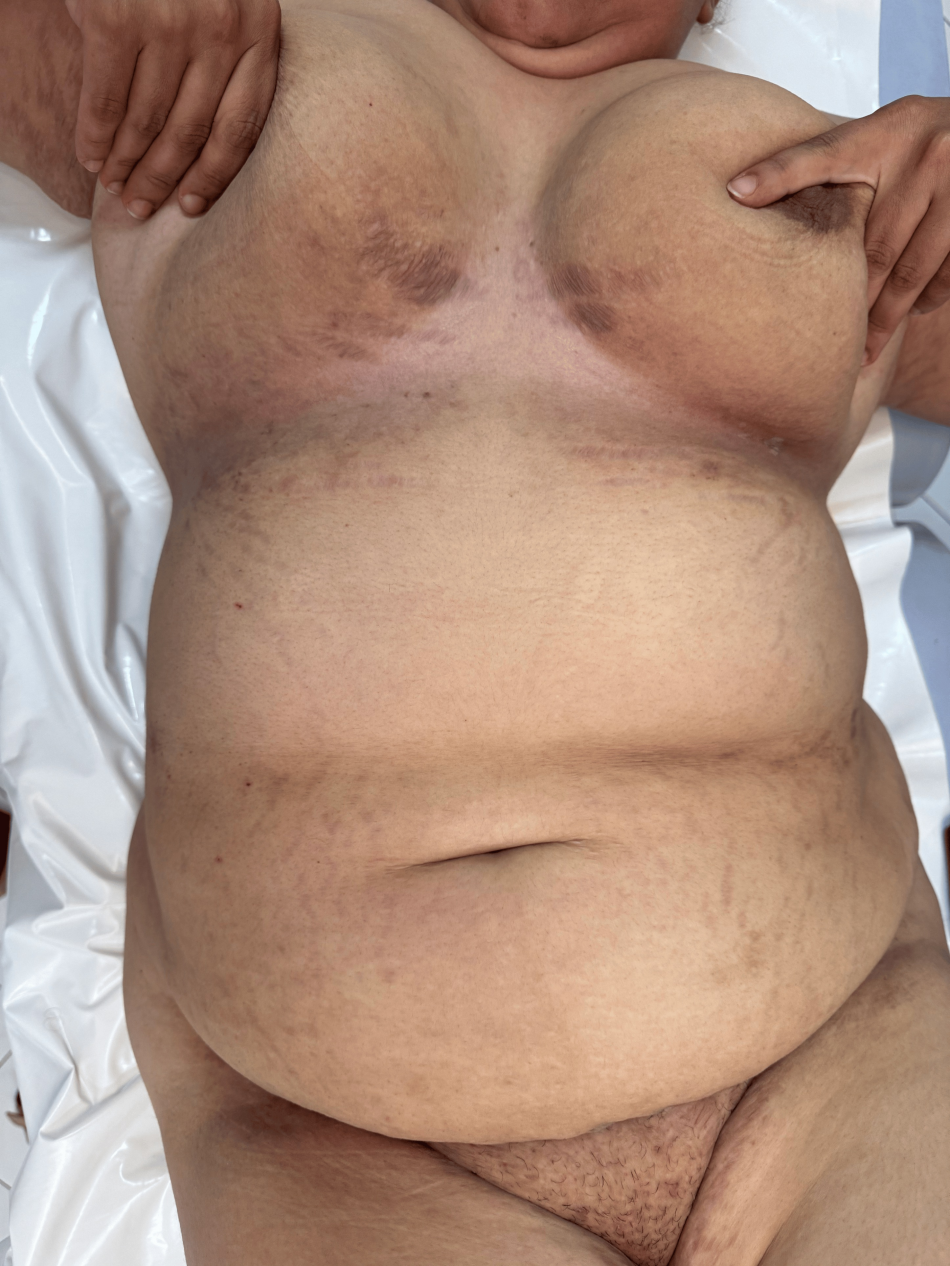
Clinical aspect after three months of treatment

Treatment included topical calcineurin inhibitors 0.1% applied twice daily to affected areas, oral doxycycline 100 mg daily for three months, and UVB phototherapy sessions twice a week. After three months of follow-up, the proband exhibited significant clinical improvement, with a reduction in erosive plaque severity, fewer flares, and improved quality of life, although the transverse leukonychia remained unchanged (Figures 4, 5).

## Discussion

HHD, or familial benign chronic pemphigus, is likely under-reported due to its variable clinical presentation and frequent misdiagnosis. Even in patients aware of a family history, subtle or atypical manifestations may be overlooked. Lesions are often non-specific at onset, but careful history-taking, including family history, can guide the correct diagnosis. Minor trauma, friction, and sweating commonly trigger lesions, particularly in the neck, axillae, groin, and inframammary folds, and patients often report significant impairment of daily activities and quality of life [4].

Nail involvement in HHD has historically been under-recognized. While Burge and colleagues reported longitudinal white bands in up to 70% of patients, these changes are usually asymptomatic, unnoticed by patients, and may precede cutaneous manifestations [2]. In contrast to Darier’s disease, nail changes in HHD are isolated, non-painful, and lack fragility, notching, red lines, or palmar pits, making them a specific early diagnostic clue [5]. Our case illustrates this principle: the proband’s solitary longitudinal leukonychia preceded the appearance of classic flexural lesions, ultimately leading to the diagnosis in multiple family members.

The mechanisms underlying nail involvement remain unknown. The distribution of lesions points toward a primary defect in the distal matrix or nail bed. Confirmatory assessment would require a biopsy of the nail matrix [6].

Histopathology remains central for diagnosis, demonstrating suprabasal acantholysis with dyskeratotic cells, often described as a “dilapidated brick wall,” while direct immunofluorescence is negative [1].

Treatment remains challenging due to the chronic, relapsing nature of HHD. Topical calcineurin inhibitors (e.g., tacrolimus 0.1%) are useful for long-term control, particularly in intertriginous areas, while oral antibiotics, especially tetracyclines, provide both anti-inflammatory and antimicrobial benefits [7]. Conventional phototherapy can reduce inflammation and flare frequency, whereas photodynamic therapy is reserved for refractory cases. Reports suggest improvement with age [2], but there is no curative therapy, and management aims to control symptoms, prevent secondary infection, and minimize triggers.

The early identification of nail changes, as highlighted in this case, can serve as a screening tool for family members and may allow timely counseling and monitoring before overt lesions develop. Our observation reinforces that nail findings in HHD are long-lasting, independent of active cutaneous disease, and can be a valuable clinical marker in both sporadic and familial cases.

## Conclusions

HHD is a chronic, acantholytic dermatosis with heterogeneous clinical expression. Nail involvement, particularly asymptomatic longitudinal leukonychia, represents a critical early diagnostic clue that can guide recognition in at-risk family members and facilitate early intervention, improving patient quality of life.
